# Assessing and Enhancing Adherence to the Standard Aseptic Non-touch Technique in Peripheral Intravenous Cannulation: A Quality Improvement Project in a Resource-Constrained Neonatal Intensive Care Unit

**DOI:** 10.7759/cureus.96730

**Published:** 2025-11-13

**Authors:** Aaisha Shahbaz, Asad Maqbool, Fahad Imami, Waiza Batool, Ahmad S Asad, Rohma Shahbaz

**Affiliations:** 1 General Surgery, Combined Military Hospital Multan, Multan, PAK; 2 Paediatrics, Combined Military Hospital Multan, Multan, PAK; 3 General Medicine, Islamic International Medical College Trust Pakistan Railway Hospital, Rawalpindi, PAK

**Keywords:** aseptic techniques, infection prevention and control, low- and middle-income country (lmic), neonatal intensive care unit (nicu), quality improvement project (qip)

## Abstract

Background

Neonatal sepsis remains a critical concern in neonatal care, contributing significantly to morbidity and mortality. Aseptic techniques prevent infections during invasive procedures such as peripheral intravenous (IV) cannulation, particularly the aseptic non-touch technique (ANTT). The purpose of this study was to assess the baseline level of ANTT adherence among medical personnel and to analyze the effect of a structured educational intervention on compliance rates. The implementation of ANTT is based on the premise that having a standard framework and ensuring its compliance will decrease infection rates and consequently improve health care, especially in the resource-constrained setups of low- and middle-income countries (LMICs)

Objectives

The primary goal of this quality improvement project (QIP) is to assess baseline adherence to the ANTT during peripheral IV cannulation and evaluate improvement following a structured educational intervention using two Plan-Do-Study-Act (PDSA) cycles in the Neonatal Intensive Care Unit (NICU) at the Combined Military Hospital, Multan, Pakistan.

Methods

This QIP comprised two PDSA cycles to improve compliance with ANTT guidelines. Baseline data on adherence to aseptic practices were collected using a QI Surveillance Proforma to measure compliance. A structured educational intervention, which included presentations, video demonstrations, hands-on training, and competency assessments, was implemented over four months to improve compliance. The target audience included the NICU staff and nursing cadets. Compliance rates were measured post-intervention in both cycles using the same standardized checklist evaluating key aseptic practices.

Results

The compliance rates for peripheral IV cannulation were initially 66%. However, the analysis post-intervention showed a significant increase in compliance, with 89% median compliance after the first PDSA cycle and 94% after the second cycle. We saw that hand hygiene improved its adherence rates from 50% to 84%, while tray cleaning practices also improved from 64% to 87%. The overall adherence rate for peripheral IV cannulation showed an increase in rates to 92%. That is a huge improvement and shows targeted educational strategies can have a substantial impact.

Conclusion

This QIP highlights that carefully planned educational interventions, training, and hands-on practice can have on NICU healthcare providers' adherence to aseptic procedures. We found that frequent skills assessments with uncomplicated, easy-to-remember procedures can ensure that our system operates near maximum efficiency.

## Introduction

Adhering to the aseptic non-touch technique (ANTT) guidelines has been proven to defend neonates against healthcare-associated infection (HCAI) due to invasive procedures, improving its control and prevention [1,2]. A lack of knowledge regarding these guidelines compromises patient care and increases rates of mortality [3]. This lack of awareness, leading to a decline in patient care, is prevalent among many healthcare setups, particularly in low- and middle-income countries (LMICs), but due to limited audits or data, there seems to be a deficiency of conclusive results available for this area.

﻿According to data published by the Centers for Disease Control and Prevention (CDC), healthcare professionals can reduce the number of HCAIs through central line-associated bloodstream infections (CLABSI) by over 70% by implementing specified aseptic measures [4]. One of the most effective ways of reducing HCAI is through the application of a standardized ANTT for a clinical procedure [5,6]. Using ANTT aims to prevent the introduction of microorganisms into susceptible body sites [2].

The core principles that ensure the ability of ANTT to improve infection prevention are the attention to key parts, key sites, and sterile areas. According to Rowley, the principles surround the idea that an aseptic field is maintained between the non-sterile equipment and the susceptible or sterile parts of the body, and ensure that only the key parts, i.e., uncontaminated parts, may establish contact with the sterile parts of the body. Healthcare organizations that use ANTT to standardize their aseptic technique report better adherence to the fundamentals of aseptic technique and decreased rates of HCAIs [6,7].

Adherence to aseptic technique, particularly in catheter practices, has been shown to reduce HCAIs across various age groups in Intensive Care Unit (ICU) settings, including neonates [1,8-11]. Therefore, the ANTT guidelines should be inculcated into the systemic framework effectively, with increased compliance in the Neonatal Intensive Care Unit (NICU) setup by increasing awareness regarding the established framework. This would improve the outcomes of neonatal admission in NICU setups. Quality improvement projects (QIPs) enable small-scale interventions that seek to reduce errors and variability [12]. This cyclical approach of planning, doing, learning, and improving continues until the intended change is realized. Plan-Do-Study-Act (PDSA) cycles have been applied and used widely in neonatal healthcare, especially to increase compliance with ANTT [13,14].

To reduce the HCAIs and to standardize the practice of peripheral intravenous (IV) cannulation, we aimed to introduce ANTT guidelines in our NICU. In our setting, peripheral IV cannulations are performed by nursing staff the majority of the time; therefore, our efforts to improve compliance were focused mainly on the nursing cadets and the present NICU nursing staff. As the guidelines formalized had not been in practice before, we used multiple PDSA cycles to familiarize the staff with them and increase their adherence to these guidelines. The aim of this QIP was to assess baseline adherence to the ANTT during peripheral IV cannulation and evaluate improvement following a structured educational intervention using two PDSA cycles, up to 80% over 12 weeks in the NICU at Combined Military Hospital (CMH) Multan. This project focused exclusively on peripheral IV cannulations performed by nursing staff in the NICU; central venous catheter insertions were not included.

## Materials and methods

This QIP comprising two cycles was conducted from April to July 2022 in the NICU of Combined Military Hospital, Multan, Pakistan. The design of the study was based on the Model for Improvement (MFI) and was guided by the PDSA cycle concept. At the beginning of this study, baseline data were collected from April 19 to May 13, 2022, in which the healthcare personnel assigned to the NICU were audited for compliance with the standard ANTT for peripheral IV cannulation.

A QI Surveillance Proforma comprising a standardized checklist, which contained predefined criteria and operational definitions to minimize subjectivity, was used to collect data on 14 peripheral IV cannulations performed by NICU nursing staff. A single trained observer carried out all assessments, ensuring consistency across observations. This approach avoided variability that might arise with multiple observers. To enhance objectivity, data were recorded in real time and based on observable behaviours. This checklist encompasses six key actions of the ANTT framework based on the ANTT guidelines. These six actions included maintaining aseptic field integrity, hand hygiene before and after key parts, cleaning key parts with sterile wipes, avoiding direct contact with sterile equipment (non-touch principle), and ensuring tray and surface decontamination before and after procedures.

After the baseline audit, the QIP team, comprising a supervisor (paediatric consultant), team leader (resident surgeon), house officers, and head nurse of the NICU, identified the gaps in the clinical practice and laid the foundation of the first PDSA cycle. The first PDSA cycle began on May 15, 2022. A plan was made to improve compliance with ANTT based on the problem areas identified in the baseline data, and an educational intervention was formalized.

Educational intervention phase of PDSA cycle 1

The educational intervention of PDSA cycle 1 was conducted from May 23 to May 27, 2022. The target audience for the educational intervention was 193 healthcare workers, comprising the NICU healthcare staff and nursing cadets. The educational intervention phase was put into place over seven days, and it included the following measures:

Training Sessions

Educational presentations and video tutorials were held on proper ANTT techniques that have been approved by the National Health Service. Hands-on practice sessions were done with all nurses and nursing students to incorporate theoretical knowledge into practical application.

Knowledge Assessment

A post-training multiple-choice questionnaire (MCQ) survey was conducted to evaluate staff comprehension and to assess the retention of ANTT principles. The MCQ exercises were conducted as part of formative educational tools to reinforce knowledge and stimulate discussion. The MCQs results were not used as an outcome measure. Staff responses were reviewed immediately during teaching sessions to provide feedback, but individual scores were not systematically collected or analyzed quantitatively.

Visual Aids

Nursing staff were given handouts that illustrated the ANTT guidelines. The posters were also displayed throughout the NICU, visible to all the healthcare staff.

Cascade Training Model

The NICU head nurse was designated as the ANTT-link nurse. She was responsible for educating her team of colleagues and junior nurses and further educating newly inducted staff regarding any ANTT-related queries. This design helped in achieving a sustainable training cascade.

Implementation Tools

ANTT guidelines were converted into printed checklists and placed in patient progress chart files attached to each NICU incubator to facilitate adherence.

Following the educational intervention, including a combination of interactive training sessions and continuous visual reminders (posters and bedside checklists), the same QI Surveillance Proforma was used to assess post-intervention adherence and healthcare workers’ compliance with ANTT protocols. A second set of 15 observational data points was collected and recorded in MS Excel (Microsoft Corporation, Redmond, Washington, United States). To analyse the data, run charts were generated using MS Excel's statistical functions to determine median adherence rates pre- and post-intervention in the first PDSA cycle.

Based on the results from the first PDSA cycle, a further action plan was made, which was continued in the second PDSA cycle. The second PDSA cycle took place from June 22, 2022, to July 21, 2022. The action plan included targeting the areas of noncompliance, and educational intervention consisted of raising awareness in the staff regarding the weaknesses, with additional emphasis on the correction of errors. Additionally, emphasis was made regarding performing the majority of the required peripheral IV cannulations of new admissions in the morning shift under supervision. A total of 25 data points were collected in the second PDSA cycle, and an analysis and an action plan were made regarding further interventions in future PDSA cycles.

During each phase of the project, we included all IV cannulation procedures that took place within the NICU of Combined Military Hospital, Multan, if they were performed by the nursing staff posted to the unit at the time. This encompassed both permanent NICU nurses and nursing cadets, as they were the target group for our educational intervention. Only those procedures that could be directly observed by the designated QI observer and documented in full using the standardized ANTT Surveillance Proforma were entered into the dataset. Cannulations carried out outside the NICU setting, such as in the operating theater or emergency department, were excluded. Similarly, procedures performed by doctors or residents were not part of the evaluation. Any cannulation event for which direct observation was not possible, or where the checklist was incomplete, was also excluded from analysis. Importantly, patient-level data such as demographics or diagnoses were not collected, as patients were not considered study subjects for this service evaluation. Similarly, to avoid overrepresentation, we did not record multiple cannulations by the same nurse within a single data collection cycle. Each cycle, therefore, included only one observed procedure per individual nurse. However, if the same staff member performed a cannulation in a subsequent cycle, those observations were included.

Although formal ethical approval was not required, as this project was done as a quality improvement and system and service analysis, ethical principles were carefully considered. Data was collected and reported at an aggregate level to protect the confidentiality of individual healthcare workers, and no personal identifiable information was recorded or shared. Participation in the educational activities was voluntary as well, and the staff were free to opt out without consequences. The project was designed to support staff in improving practice without imposing additional workload burdens. Patient data was not included in this service evaluation.

Statistical analysis

As this was a pragmatic, feasibility-driven QI project, the sample sizes for observed cannulations were determined by the number of eligible procedures that occurred during the respective observation windows and staff availability for direct observation. The total sample size was 14 at baseline, 15 in the first PDSA cycle, and 25 in the second. The aim was to collect at least 10 data points per phase to allow detection of non-random signals, i.e., shifts or trends, in time-ordered data while extrapolating these data points on run charts where each data point represented one episode of peripheral IV cannulation. Run charts were chosen to detect any change in adherence to ANTT over the course of our PDSA cycles, as they are widely accepted in QI methodology and can compare the performance measure before and after intervention in real time. Interpretation of a run chart can aid in the identification of any non-random variation, i.e., shifts (six or more consecutive points above or below the central line) or trends (five or more consecutive points all going up or down). 

## Results

The QIP was aimed at improving adherence to the ANTT. It produced noteworthy results regarding the rate of compliance before and after the intervention. The results for the project were categorized into the baseline and post-intervention adherence rates.

Baseline adherence rates

Before we initiated the educational intervention, the baseline data were collected to assess adherence to ANTT during cannulation procedures, which was observed to be recorded at 66%. The baseline results before the intervention indicated poor adherence to key procedural and hygiene protocols. The compliance rates for the initial handwashing were found to be only 50%, which highlighted room for improvement in the infection control practices taken by all healthcare staff of the NICU. It was observed that thoroughly cleaning the tray before use was performed in only 64% of cases, while washing hands before unpacking equipment was absent and was recorded to be 0%. We observed that washing hands before putting on gloves was 21%, and the compliance rates for the final hand wash were only 57%. We also noted that cleaning the tray after use was documented at 36%.

First PDSA cycle adherence rates

The post-intervention data indicated a marked improvement in compliance across various practices, and an increase in compliance rates with the protocols was observed. The median adherence rate for peripheral IV cannulation practices increased to 89% after the intervention. This reflected a significant improvement in the application of ANTT principles. Initial handwashing compliance improved from the original 50% at baseline to 87% post-intervention, while cleaning the tray before use increased from 64% to 87%. A particularly noteworthy improvement was seen in washing hands before unpacking equipment, which had 0% compliance at baseline but reached 67% post-intervention, indicating a substantial change.

This growing awareness of improved standards to maintain asepsis was also observed when washing hands before wearing gloves was shown to have increased from 21% to 73%. There were further improvements observed as it was evident that cleaning the tray after use showed a better adherence from 36% to 60%, and final handwashing from 57% to 87%. These findings proved that the intervention conducted had successfully introduced compliance with essential aseptic protocols.

**Figure 1:**
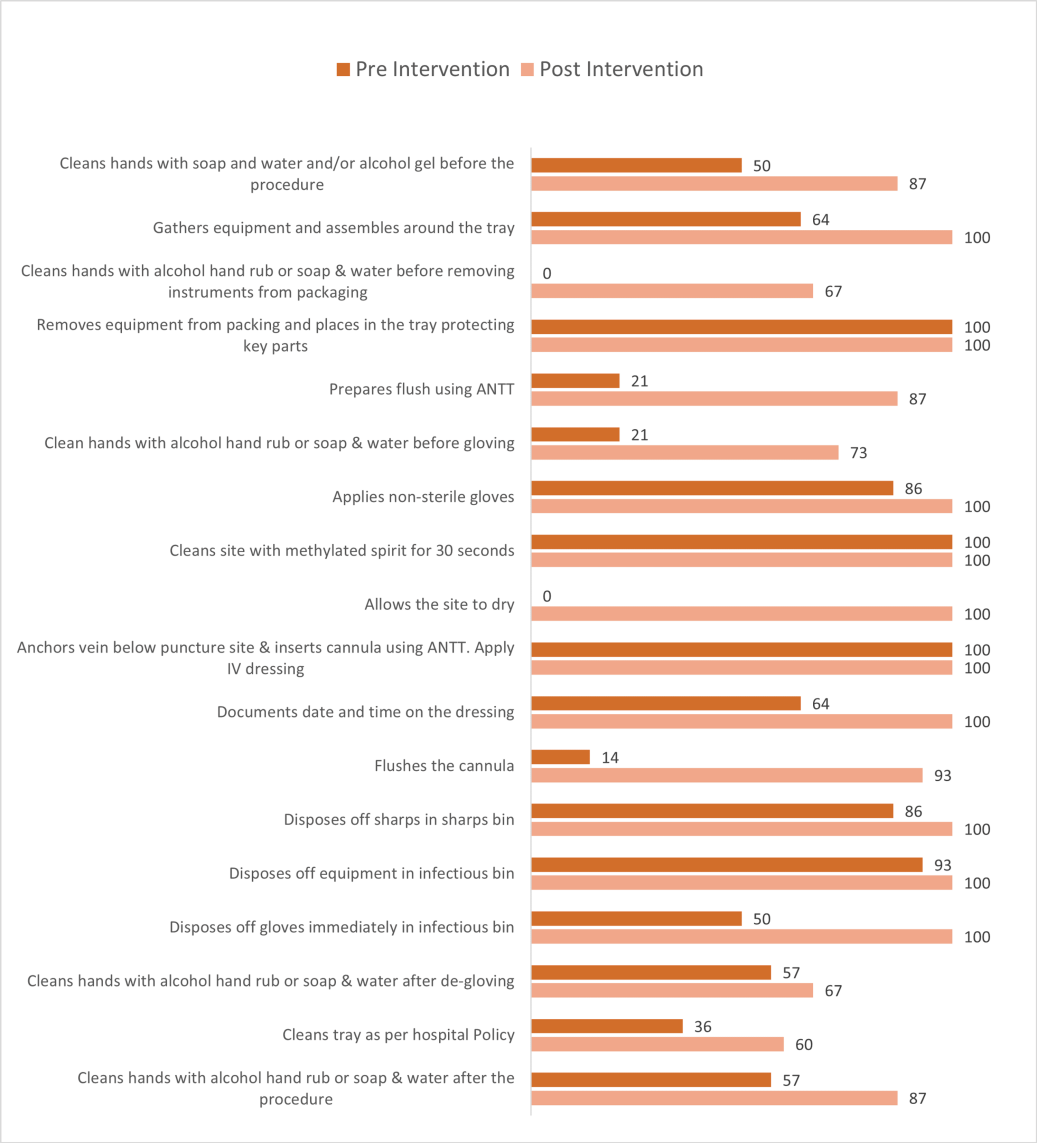
Comparison of aseptic non-touch technique compliance pre- and post-educational intervention in intravenous cannulation

The run chart showed that the initial levels of compliance ranged from 40% to 80% and the median adherence of only 66%. A significant improvement in adherence was observed after the first intervention, with compliance rates stabilizing at a higher range (80%-100%). The median compliance increased to 89%, demonstrating the effectiveness of the initial quality improvement measures, such as training sessions, reminders, or audits. Some variations in compliance were still present, which were indicators that further reinforcement might be needed. Figure 1 shows the adherence to each step of the IV cannulation checklist according to the ANTT pre- versus post-educational intervention.

Second PDSA cycle adherence rates

**Figure 2:**
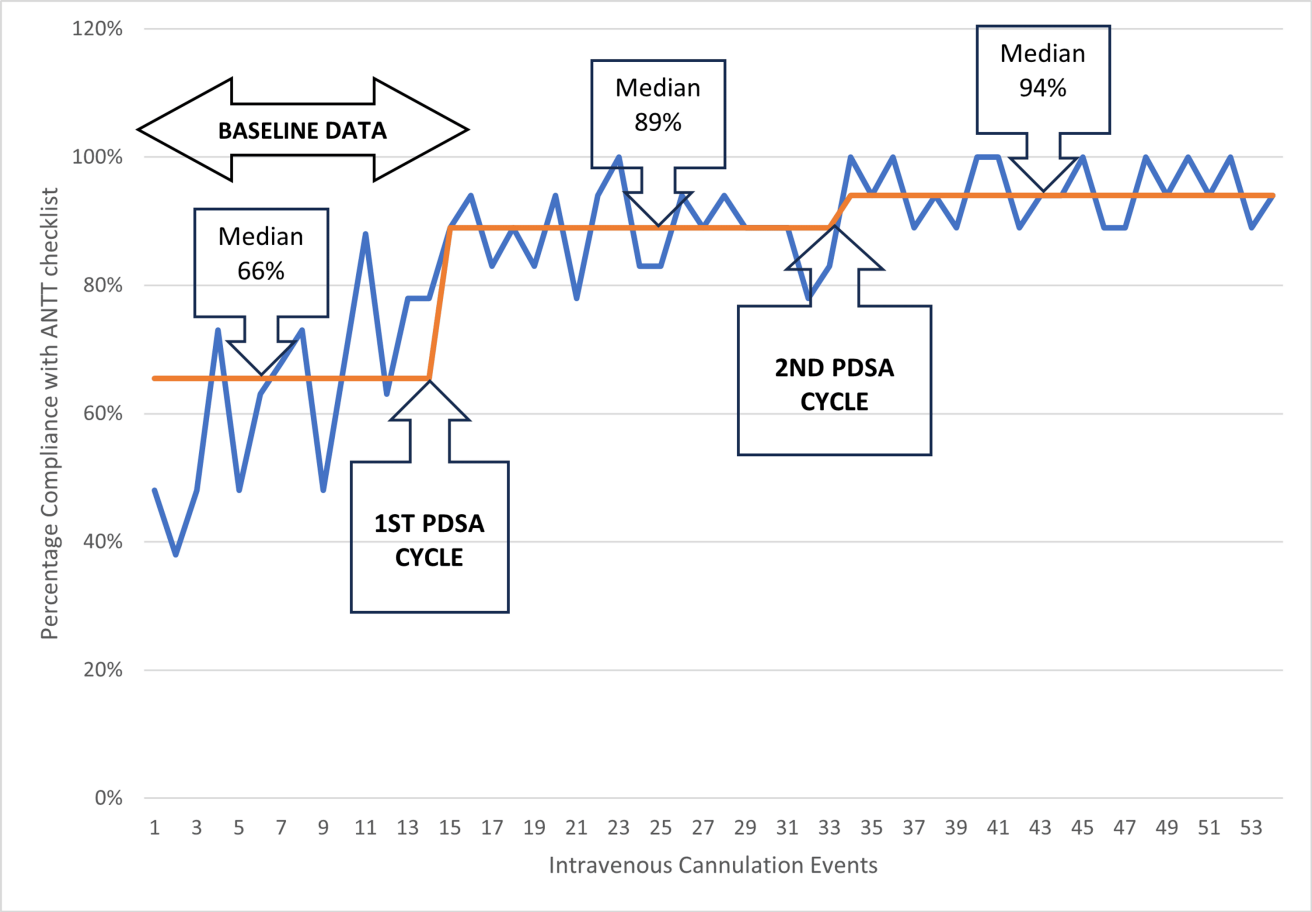
Percentage adherence to aseptic non-touch technique in intravenous cannulation

The second PDSA cycle showed remarkable improvement in the ANTT adherence rates, with only one IV cannulation data point showing 78% adherence and eight events showing 100% adherence, allowing the median to shift from 89% to 94%. Figure 2 shows the run chart displaying the data points of baseline adherence, first and second PDSA cycles, and the median of each cycle.

## Discussion

Neonatal sepsis is one of the main threats to newborn survival, and the problem is particularly severe in countries where healthcare resources are stretched thin. By bringing in ANTT, evidence shows that HCAIs are significantly reduced. Shettigar and colleagues documented how introducing ANTT in a NICU setting brought about a marked drop in HCAI rates. Their approach, which relied on structured educational activities much like those in our quality improvement initiative, strengthened adherence to aseptic protocols and, in turn, reduced infection risk [13].

The CDC has long pointed out the importance of following standard aseptic methods, especially when it comes to protecting vulnerable newborns. Their guidelines highlight that adhering closely to these protocols, like ANTT, really helps reduce bloodstream infections. This is particularly important in NICUs, where babies’ immune systems are still developing and have reduced ability to ward off infections [4]. In practice, success with ANTT depends on robust clinical governance that takes ownership of infection control, systems that hold staff accountable, and regular audits to keep standards from slipping [14].

When we initiated ANTT in our NICU, we saw a noticeable improvement in how consistently staff followed the technique. Our baseline data suggested that compliance with aseptic principles for peripheral IV cannulation in our NICU was only about two-thirds. These figures emphasized that there were gaps in maintaining hygiene and following protocols. This potentially increased the risk of contamination and infection. These observations further reinforced the need for intervention and enhanced adherence to protocols.

The results demonstrated that our intervention effectively addressed certain areas that showed trends of non-compliance. The most significant improvements were noted to have been observed in the protocols concerned with the healthcare workers' hand hygiene. The increase in adherence to hand hygiene in the initial handwash indicates an improvement in the staff behavior toward more effective infection control practices. Before the intervention, there was 0% adherence rate for cleaning hands with alcohol hand rub or soap and water after gathering the equipment and before coming in contact with the clean equipment, which showed a significant increase to 67% after the first PDSA cycle. This change signifies the staff's improved awareness concerning hand hygiene and Implementation of aseptic protocols.

In our NICU, we wanted to go beyond signposting guidelines. Therefore, we initiated a hands-on educational program that combined brief talks, practical training, and competency checks. Our goal was to make sure the staff not only understood the theory but could also confidently apply these steps during their daily work. Guerrero-Díaz and colleagues observed that classroom-based teaching on its own rarely equips nurses to conduct ANTT effectively when preparing medications. In their work, they moved beyond theory by adding tailored, one-on-one practical sessions [15]. At first, the approach leaned heavily on the nurses’ prior experience, but the addition of supervised, hands-on training noticeably boosted their confidence and skill in applying the technique. Interestingly, Sonoiki et al. reported that even seasoned nurses can feel uneasy when asked to adopt methods they have never used before [16]. For this reason, the World Health Organization advises that both theoretical instruction and practical application should be built into training for new clinical procedures. In the UK, the Health and Social Care Act 2012 also underlines the need to monitor how well such techniques are followed in day-to-day care [17].

Balachander’s study shows that setting up a steady training routine and a checklist for inserting peripheral IV lines is not only doable but also sustainable. Their initial training led to a noticeable drop in bloodstream infections, and awareness around the practice has been steadily growing since. Although the improvements we saw in compliance rates are promising, reaching full adherence, i.e., 100%, still needs to be achieved. Future efforts should aim at not just maintaining but building on these gains by continuing staff training, conducting regular audits, and reinforcing best practices. This is in line with the recommendations by Clare and Rowley. They emphasize the need to weave ANTT guidelines firmly into the healthcare system’s structure to achieve lasting progress in preventing infections [11].

We find it important to also acknowledge limitations in this study. Even though we saw a clear improvement in compliance rates, it will be a challenge to maintain these improvements over time. To maintain high compliance, it is essential to provide ongoing assessments, regular training, and foster a culture of safety. Future studies should focus on long-term follow-up to see how lasting these improvements are and how they affect the key outcomes, like rates of newborn sepsis. Additionally, while this project offers useful insights into how well the ANTT framework works in our NICU, it is important to remember that it took place in a single hospital. Other hospitals, especially in LMICs, may face different challenges when it comes to staff training and resources; therefore, the results might not be reproducible in other settings. Additionally, this project addressed only the insertion phase of peripheral IV cannulation. Subsequent care, maintenance, and replacement of catheters, although equally critical for preventing infection, were outside the project’s scope. This project did not measure patient-level outcomes such as infection or sepsis rates; however, adherence to aseptic practice has been well-documented to reduce HCAIs. Future QIPs should incorporate direct monitoring of clinical outcomes to establish a measurable impact on neonatal sepsis rates.

Bringing ANTT into everyday practice using these quality improvement models helps in keeping clinical practice safe and can cut down on HCAIs over time. These cycles allowed us to observe the aseptic practices and make changes if required [1,13]. Thorough training on the ANTT framework is important for healthcare staff to make sure it is done right [14]. Overall, this QIP underlines how valuable structured educational programs are for helping NICU staff stick to aseptic procedures. By following ANTT guidelines carefully, we have a real opportunity to reduce infections in newborns and improve patient outcomes.

## Conclusions

This QI project emphasized that a structured ANTT training program of standard guidelines improves the practice of ANTT in healthcare professionals. We saw quantifiable improvements in areas like handwashing and cleaning equipment, which showed that regular education and training make a real difference in adherence to guidelines.

In this project, we also realized the importance of embedding continuous training, regular monitoring, and visible reminders within daily clinical activities to sustain change and strengthen the culture of patient safety. Although this project was limited due to small sample sizes, the results provide a strong rationale for scaling up ANTT-focused education and surveillance across other units and for incorporating ANTT compliance into routine clinical practice.
